# The relationship between serum creatinine-to-albumin ratio (CAR) and prognosis in critically ill patients with acute heart failure admitted to the ICU: A retrospective study based on the MIMIC-IV database

**DOI:** 10.1097/MD.0000000000046825

**Published:** 2026-01-09

**Authors:** Shenglan Liu, Chengyu Zhang, Yuanxi Feng

**Affiliations:** a Department of Critical Care Medicine, The First Affiliated Hospital of Soochow University, Suzhou, China; b Wuxi School of Medicine, Jiangnan University, Wuxi, China; c Department of Cardiovascular Medicine, Jiangnan University Medical Center, Wuxi, China.

**Keywords:** acute heart failure, creatinine-to-albumin ratio, ICU, intensive care unit, MIMIC-IV database, mortality risk, retrospective study

## Abstract

Acute heart failure (AHF) remains associated with high short- and long-term mortality, particularly in patients admitted to the intensive care unit (ICU). Reliable biomarkers for early risk stratification are urgently needed. The creatinine-to-albumin ratio (CAR), integrating renal function and nutritional–inflammatory status, may serve as a novel prognostic marker in critically ill AHF patients. We conducted a retrospective cohort analysis using the Medical Information Mart for Intensive Care IV database, including 7620 patients with a 1st ICU admission for AHF. CAR was calculated from serum creatinine and albumin measured at ICU admission. The primary endpoint was 28-day all-cause mortality; secondary endpoints included 180-day and 365-day all-cause mortality. Survival outcomes were analyzed using Kaplan–Meier curves, Cox proportional hazards regression models, and restricted cubic spline analyses. Patients with higher CAR levels had significantly higher 28-, 180-, and 365-day mortality (all log-rank *P* < .001). In multivariate Cox models, elevated CAR was independently associated with increased mortality, and this association was consistent across all adjustment models. Restricted cubic spline analyses confirmed a nonlinear dose–response relationship between CAR and short- and long-term mortality risk. Subgroup analyses demonstrated consistent associations across most patient subgroups, without significant interactions. CAR is a simple and accessible prognostic biomarker that independently predicts short- and long-term mortality in critically ill patients with AHF admitted to the ICU. Incorporating CAR into clinical practice may improve early risk stratification and guide management decisions. Prospective studies are warranted to validate its clinical utility.

**Key points**

- Utilized data from the large-scale MIMIC-IV database, ensuring a sufficiently large sample size (7620 patients) to enhance the statistical robustness of the findings.
- Employed multiple rigorous statistical methods (Kaplan–Meier curves, Cox regression, and restricted cubic spline models) and adjusted for extensive covariates, improving the reliability of the association between CAR and mortality risk.
- Evaluated both short-term (28-day) and long-term (180-day, 365-day) mortality endpoints, providing comprehensive insights into CAR’s prognostic value across different timeframes.
- Retrospective study design may introduce selection bias, as data were derived from a single-center database (Beth Israel Deaconess Medical Center), potentially limiting generalizability to other populations.
- Relied on baseline CAR measurements only, lacking dynamic monitoring of CAR changes during hospitalization, which could affect the assessment of its real-time prognostic utility.

## 1. Introduction

Heart failure (HF) represents a significant global public health burden, affecting over 64 million individuals worldwide. Acute heart failure (AHF), the most severe manifestation of HF, is characterized by high mortality rates and frequent hospitalizations.^[1]^ Studies report that AHF patients face 1-year mortality rates approaching 40%,^[2]^ imposing substantial physical and psychological burdens on patients and families while creating significant economic strain on healthcare systems. This challenge is particularly critical in intensive care unit (ICU) settings where AHF patients frequently present with multi-organ dysfunction, limiting the prognostic utility of traditional markers such as left ventricular ejection fraction for risk stratification.^[3]^ Consequently, developing rapid and accessible biomarkers to improve risk assessment and therapeutic decision-making in critically ill AHF patients constitutes an urgent research priority.

In addition to left ventricular ejection fraction, several traditional markers and risk scores have been widely applied in the prognostic assessment of AHF patients. Biomarkers such as B-type natriuretic peptide (BNP) and N-terminal proBNP (NT-proBNP) are well-established indicators of cardiac stress and have strong prognostic implications in both AHF and chronic heart failure.^[4,5]^ Similarly, cardiac troponins reflect myocardial injury and are independently associated with adverse outcomes, including 1-year mortality and rehospitalization.^[6]^ Beyond single biomarkers, validated risk models such as Acute Decompensated Heart Failure National Registry and Organized Program to Initiate Lifesaving Treatment in Hospitalized Patients with Heart Failure provide structured tools for outcome prediction in hospitalized HF populations, though their applicability in ICU settings may be limited.^[7]^ These limitations highlight the need for novel, integrative prognostic indicators. Renal impairment and nutritional status serve as pivotal determinants of HF prognosis.

Renal impairment and nutritional status serve as pivotal determinants of HF prognosis. Serum creatinine, the cornerstone metric for estimating glomerular filtration rate, exhibits limited reliability in acute settings due to susceptibility to fluid status fluctuations, muscle mass variations, and hemodynamic instability.^[3]^ Conversely, serum albumin functions as both a nutritional indicator and biomarker of systemic inflammation. Hypoalbuminemia independently predicts elevated mortality, with Polat et al documenting 1-year mortality rates of 37% versus 12% in patients with albumin levels below versus above 3.10 g/dL.^[8]^ These findings underscore the clinical need for integrated biomarkers that concurrently reflect renal function and inflammatory–nutritional status.

The creatinine-to-albumin ratio (CAR) has emerged as a novel composite biomarker with unique pathophysiological relevance. By simultaneously quantifying renal dysfunction through serum creatinine and systemic inflammation coupled with nutritional depletion through serum albumin, CAR provides a holistic assessment of disease severity. Its prognostic value has been validated across diverse clinical contexts including acute kidney injury where elevated CAR correlates with increased mortality risk,^[9]^ oncology populations where it predicts survival outcomes,^[10]^ and stroke cohorts with similar prognostic associations.^[11]^

Notably, a significant knowledge gap persists regarding CAR’s prognostic significance specifically in critically ill AHF populations. This unmet research need underscores the novelty of our investigation, which offers a clinically accessible CAR dual-parameter assessment tool to address current limitations in risk stratification. Leveraging the Medical Information Mart for Intensive Care IV (MIMIC-IV) database,^[12]^ a comprehensive repository of high-fidelity ICU patient data, this study aims to establish CAR’s independent prognostic value in ICU-admitted AHF patients, characterize its dose–response relationship with mortality risk, and determine its incremental utility beyond established prognostic factors. These insights may inform early identification of high-risk individuals and optimize clinical management pathways.

## 2. Materials and methods

### 2.1. Data source and study population

**Figure 1.**
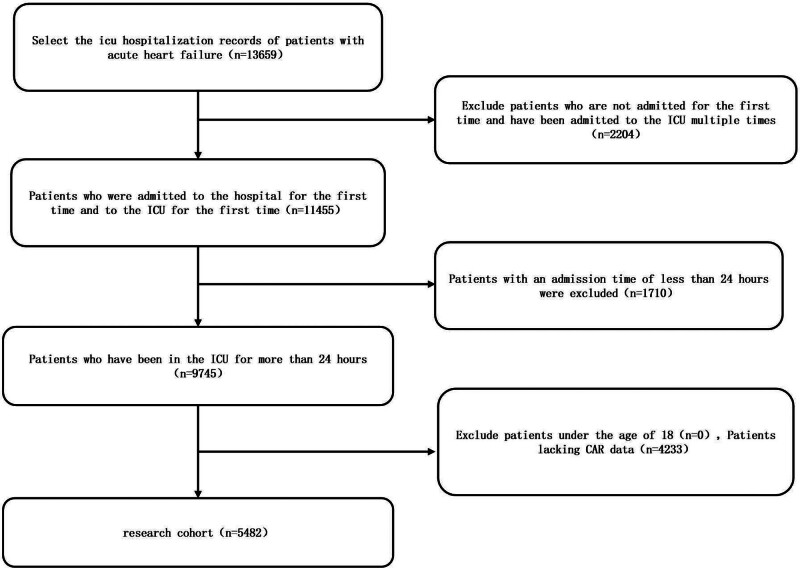
Flowchart of patient selection for the study cohort. Flowchart illustrating the selection process for patients with acute heart failure (AHF) admitted to the intensive care unit (ICU) from the MIMIC-IV database. Among 13,659 ICU admissions, patients with repeated ICU stays (n = 2204), admission duration <24 hours (n = 1710), age under 18 years (n = 0), or missing creatinine-to-albumin ratio (CAR) data (n = 4233) were excluded. A total of 5482 patients met the inclusion criteria and were included in the final analysis. MIMIC-IV = Medical Information Mart for Intensive Care IV.

We conducted a retrospective cohort analysis using clinical data extracted from the medical information from MIMIC-IV database, a publicly available repository containing de-identified health records of over 50,000 critically ill patients admitted to Beth Israel Deaconess Medical Center between 2008 and 2019. Data access authorization to MIMIC-IV was certified by Chengyu Zhang (CITI record No. 13849257). Among the initial 11,455 patients, the inclusion criteria we applied included: diagnosis of AHF based on ICD-9/ICD-10 coding; 1st hospitalization and 1st admission to the ICU. Exclusion criteria included ICU stay duration <24 hours and missing creatinine or albumin at admission (n = 3825). The final analysis cohort included 7620 patients stratified by CAR levels, with the final cohort selection process illustrated in Figure 1.

### 2.2. Study variables

The CAR ratio was calculated based on the 1st laboratory test data after admission, using the following formula: CAR = serum creatinine concentration (mg/dL) ÷ serum albumin concentration (g/dL). The primary endpoint was 28-day all-cause mortality, with secondary endpoints including 180- and 365-day all-cause mortality.

### 2.3. Covariates

Covariates were extracted from the MIMIC-IV database using PostgreSQL (version 17.2) and included the following: demographic variables (age, gender, etc); laboratory parameters (white blood cell count [WBC], red blood cell count [RBC], platelet count, hemoglobin, red blood cell distribution width [RDW], hematocrit, electrolytes including sodium, potassium, chloride, calcium, coagulation markers such as prothrombin time [PT] and international normalized ratio [INR], renal function markers such as blood urea nitrogen [BUN] and creatinine); disease severity scores (Sequential Organ Failure Assessment [SOFA], Acute Physiology Score III [APS III], Oxford Acute Severity of Illness Score [OASIS], and Systemic Inflammatory Response Syndrome [SIRS]); comorbidities (hypertension, diabetes, chronic obstructive pulmonary disease [COPD], myocardial infarction, atrial fibrillation, cerebrovascular disease, dyslipidemia, liver dysfunction, malignant tumors, coronary heart disease, chronic kidney disease, cardiomyopathy); drug therapy (angiotensin-converting enzyme inhibitors (ACEIs), angiotensin receptor blockers [ARBs], diuretics, statins, antiplatelet agents such as aspirin and clopidogrel, anticoagulants such as warfarin and direct oral anticoagulants); and interventional treatments (coronary artery bypass grafting [CABG], PCI, and implantable cardioverter-defibrillator implantation). Continuous variables with missing data exceeding 20% were excluded; variables with <20% missing data were imputed using the *k*-nearest neighbor interpolation method in the DMwR2 package of R language.

### 2.4. Statistical analysis

Categorical variables were expressed as frequencies and percentages, and intergroup comparisons were performed using chi-square tests. Continuous variables were expressed as means ± standard deviations, and comparisons were performed using *t* tests or analysis of variance. Survival differences were assessed using Kaplan–Meier curves based on CAR quartiles, and statistical comparisons were performed using log-rank tests. The association between CAR and mortality was assessed using Cox proportional hazards models. After validating the proportional hazards assumption using Schoenfeld residuals and ensuring that multicollinearity (variance inflation factor < 5) was controllable, 4 adjusted models were constructed: the unadjusted model included only CAR; the baseline-adjusted model adjusted for age and sex; the comorbidity-adjusted model further adjusted for hypertension, diabetes, COPD, myocardial infarction, atrial fibrillation, cerebrovascular disease, dyslipidemia, liver dysfunction, malignant tumors, coronary artery disease, chronic kidney disease, and cardiomyopathy; the fully adjusted model further adjusted for laboratory parameters (WBC count, RBC count, platelet count, hemoglobin, RDW, hematocrit, sodium, potassium, chloride, calcium, PT, INR, BUN, estimating glomerular filtration rate), disease severity scores (SOFA, APS III, OASIS, SIRS), drug therapy (ACEIs/ARB, diuretics, statins, antiplatelet agents, anticoagulants), and interventional treatments (CABG, PCI, implantable cardioverter-defibrillator). Restricted cubic spline (RCS) analysis was used to describe the nonlinear relationship between CAR and mortality, and subgroup analysis (stratified by age, gender, comorbidities, etc) was conducted to validate the robustness of the results. All statistical analyses were performed using R software (version 4.1.3; R Foundation for Statistical Computing, Vienna, Austria), and a 2-sided *P*-value < .05 was considered statistically significant.

## 3. Results

### 3.1. Baseline characteristics

Table 1 presents the baseline characteristics of 7620 critically ill patients with AHF from the MIMIC-IV database, categorized into quartiles based on the CAR measured at ICU admission. The cutoff values were as follows: Group 1 (Q1): 0.05 to 0.30 (n = 1905), Group 2 (Q2): 0.30 to 0.44 (n = 1905), Group 3 (Q3): 0.44 to 0.72 (n = 1905), Group 4 (Q4): 0.72 to 8 (n = 1905). The overall cohort had a mean age of 71.96 years (SD = 13.67), with males comprising 54.8% (n = 4172). Patients in the higher CAR quartile (Q4) exhibited significant differences compared to lower quartiles. Laboratory parameters: Q4 patients had significantly higher WBC, RDW, anion gap, PT, partial thromboplastin time, INR, BUN, and creatinine levels (all *P* < .001). Conversely, they had significantly lower RBC count, platelet count, hemoglobin, sodium, chloride, lymphocyte count, and albumin levels (all *P* < .001). Disease severity: scores for SOFA, APS III, and OASIS were significantly elevated in Q4 (all *P* < .001). The distribution of SIRS scores also differed significantly across quartiles (*P* = .003). Comorbidities: the prevalence of diabetes mellitus, atrial fibrillation, myocardial infarction, COPD, liver disease, and renal disease was significantly higher in Q4 (all *P* < .001). Hypertension and dyslipidemia prevalence decreased significantly with increasing CAR quartile (both *P* < .001). Interventions and outcomes: the use of diuretics, ACEIs/ARBs, PCI, and CABG varied significantly across quartiles (all *P* < .001). Total hospital length of stay and ICU length of stay were significantly longer in Q4 (both *P* < .001). Critically, 28-, 180-, and 365-day all-cause mortality rates were markedly higher in Q4 (all *P* < .001). In contrast, the prevalence of cerebrovascular disease and malignant cancer, as well as the 180-day readmission rate, did not differ significantly across CAR quartiles (*P* = .919, *P* = .359, and *P* = .236, respectively).

**Table 1:** Baseline characteristics of participants by creatinine-to-albumin ratio (CAR) quartiles.

	Total (n = 7620)	CAR quartiles	Group 2 (n = 1905)	Group 3 (n = 1905)	Group 4 (n = 1905)	*P*-value
		Group 1 (n = 1905)				
Age (yr)	71.96 (13.67)	70.30 (14.11)	72.81 (13.74)	73.86 (13.20)	70.85 (13.32)	<.001
Gender n (%)						<.001
Male	4172 (54.8)	757 (39.7)	1076 (56.5)	1103 (57.9)	1236 (64.9)	
Female	3448 (45.2)	1148 (60.3)	829 (43.5)	802 (42.1)	669 (35.1)	
Length of stay (LOS) (d)	15.32 (13.89)	13.48 (12.59)	15.00 (12.55)	15.69 (13.70)	17.13 (16.17)	<.001
ICU length of stay (ICULOS) (d)	5.35 (6.10)	4.71 (5.19)	5.24 (5.93)	5.47 (6.34)	5.96 (6.78)	<.001
WBC (K/μL)	12.29 (8.08)	11.81 (6.63)	12.08 (7.82)	12.85 (9.52)	12.42 (8.06)	<.001
RBC (m/μL)	3.54 (0.80)	3.73 (0.80)	3.65 (0.81)	3.52 (0.80)	3.27 (0.73)	<.001
Platelet (K/μL)	217.59 (99.69)	229.81 (102.52)	215.66 (96.11)	214.84 (98.89)	210.04 (100.12)	<.001
RDW (%)	15.98 (2.48)	15.28 (2.27)	15.73 (2.42)	16.27 (2.51)	16.65 (2.49)	<.001
Hemoglobin (g/dL)	10.37 (2.49)	10.94 (2.30)	10.70 (2.29)	10.27 (3.02)	9.56 (2.01)	<.001
Sodium (mmol/L)	137.32 (4.37)	137.61 (4.08)	137.66 (4.26)	137.34 (4.54)	136.69 (4.51)	<.001
Potassium (mmol/L)	4.25 (0.60)	4.09 (0.54)	4.18 (0.56)	4.28 (0.59)	4.46 (0.63)	<.001
Chloride (mmol/L)	100.82 (6.50)	101.45 (6.15)	101.59 (5.86)	100.93 (6.61)	99.31 (7.05)	<.001
Aniongap (mmol/L)	15.26 (4.11)	13.48 (3.27)	14.33 (3.33)	15.51 (3.84)	17.70 (4.57)	<.001
PT (s)	18.49 (10.58)	16.22 (7.39)	17.93 (9.12)	19.75 (11.47)	20.07 (13.00)	<.001
PTT (s)	36.41 (12.90)	35.16 (12.53)	36.17 (13.10)	36.91 (13.07)	37.40 (12.80)	<.001
INR	1.62 (0.78)	1.46 (0.59)	1.59 (0.70)	1.72 (0.90)	1.71 (0.87)	<.001
Lymphocyte count (K/μL)	12.24 (9.22)	13.96 (9.54)	12.94 (9.36)	11.46 (9.19)	10.63 (8.38)	<.001
BUN (mg/dL)	38.53 (26.40)	18.59 (8.34)	28.23 (13.07)	42.20 (19.71)	65.08 (30.61)	<.001
CRE (mg/dL)	1.91 (1.62)	0.79 (0.20)	1.19 (0.23)	1.74 (0.38)	3.92 (2.10)	<.001
Albumin (g/dL)	3.24 (0.60)	3.51 (0.58)	3.28 (0.56)	3.14 (0.57)	3.02 (0.60)	<.001
SOFA	5.50 (3.28)	3.89 (2.86)	4.87 (2.89)	5.85 (3.16)	7.37 (3.14)	<.001
APSIII	50.00 (18.17)	40.19 (15.13)	45.44 (15.41)	53.91 (16.87)	60.48 (18.12)	<.001
OASIS	32.66 (8.40)	31.79 (7.87)	32.23 (7.96)	33.05 (8.55)	33.59 (9.07)	<.001
SIRS, n (%)						
0	44 (0.6)	8 (0.4)	14 (0.7)	10 (0.5)	12 (0.6)	.003
1	895 (11.7)	207 (10.9)	209 (11.0)	240 (12.6)	239 (12.5)	
2	2614 (34.3)	673 (35.3)	668 (35.1)	616 (32.3)	657 (34.5)	
3	3002 (39.4)	777 (40.8)	784 (41.2)	736 (38.6)	705 (37.0)	
4	1065 (14.0)	240 (12.6)	230 (12.1)	303 (15.9)	292 (15.3)	
Hypertension, n (%)	1588 (20.8)	690 (36.2)	500 (26.2)	270 (14.2)	128 (6.7)	<.001
Diabetes, n (%)	3501 (45.9)	619 (32.5)	811 (42.6)	950 (49.9)	1121 (58.8)	<.001
Atrial fibrillation, n (%)	4173 (54.8)	946 (49.7)	1065 (55.9)	1109 (58.2)	1053 (55.3)	<.001
Dyslipidemia, n (%)	3700 (48.6)	862 (45.2)	958 (50.3)	972 (51.0)	908 (47.7)	.001
Myocardial infarct, n (%)	2746 (36.0)	602 (31.6)	686 (36.0)	697 (36.6)	761 (39.9)	<.001
Cerebrovascular disease, n (%)	908 (11.9)	228 (12.0)	234 (12.3)	220 (11.5)	226 (11.9)	.919
COPD, n (%)	2958 (38.8)	815 (42.8)	744 (39.1)	726 (38.1)	673 (35.3)	<.001
Liver disease, n (%)	829 (10.9)	156 (8.2)	174 (9.1)	207 (10.9)	292 (15.3)	<.001
Renal disease, n (%)	3547 (46.5)	144 (7.6)	634 (33.3)	1194 (62.7)	1575 (82.7)	<.001
Malignant cancer, n (%)	808 (10.6)	202 (10.6)	207 (10.9)	216 (11.3)	183 (9.6)	.359
Coronary artery disease, n (%)	3540 (46.5)	825 (43.3)	905 (47.5)	911 (47.8)	899 (47.2)	.016
Cardiomyopathy, n (%)	1817 (23.8)	388 (20.4)	478 (25.1)	488 (25.6)	463 (24.3)	<.001
Implantable cardioverter defibrillator pacemaker, n (%)	414 (5.4)	115 (6.0)	124 (6.5)	99 (5.2)	76 (4.0)	.004
Percutaneous coronary intervention, n (%)	915 (12.0)	299 (15.7)	275 (14.4)	195 (10.2)	146 (7.7)	<.001
Coronary bypass operation, n (%)	532 (7.0)	208 (10.9)	162 (8.5)	91 (4.8)	71 (3.7)	<.001
Used diuretic, n (%)	6965 (91.4)	1798 (94.4)	1823 (95.7)	1826 (95.9)	1518 (79.7)	<.001
Used antiplatelet, n (%)	5502 (72.2)	1313 (68.9)	1422 (74.6)	1377 (72.3)	1390 (73.0)	.001
Used anticoagulant, n (%)	7256 (95.2)	1777 (93.3)	1802 (94.6)	1824 (95.7)	1853 (97.3)	<.001
Used statin, n (%)	4921 (64.6)	1184 (62.2)	1260 (66.1)	1225 (64.3)	1252 (65.7)	.044
Used ACEI/ARB, n (%)	3459 (45.4)	1079 (56.6)	1046 (54.9)	808 (42.4)	526 (27.6)	<.001
180-day readmission, n (%)	1959 (25.7)	464 (24.4)	504 (26.5)	478 (25.1)	513 (26.9)	.236
CAR (mean (SD))	0.61 (0.55)	0.23 (0.05)	0.36 (0.04)	0.56 (0.08)	1.31 (0.70)	<.001
Death within 28 d, n (%)	1221 (16.0)	165 (8.7)	239 (12.5)	350 (18.4)	467 (24.5)	<.001
Death within 180 d, n (%)	2456 (32.2)	370 (19.4)	520 (27.3)	698 (36.6)	868 (45.6)	<.001
Death within 365 d, n (%)	3049 (40.0)	475 (24.9)	658 (34.5)	852 (44.7)	1064 (55.9)	<.001

Continuous variables are presented as mean ± standard deviation (SD), and categorical variables are expressed as number (%).

APS III = Acute Physiology Score III; BUN = blood urea nitrogen; CAR = creatinine-to-albumin ratio; COPD = chronic obstructive pulmonary disease; INR = international normalized ratio; OASIS = Oxford Acute Severity of Illness Score; PT = prothrombin time; PTT = partial thromboplastin time; RBC = red blood cell count; RDW = red blood cell distribution width; SIRS = Systemic Inflammatory Response Syndrome; SD = standard deviation; SOFA = Sequential Organ Failure Assessment; WBC = white blood cell count.

Table 2 compares baseline characteristics between 28-day survivors (n = 6399) and non-survivors (n = 1221). Non-survivors had a significantly higher mean CAR level (0.76 vs 0.59, *P* < .001). They were also significantly older (mean 76.48 vs 71.09 years, *P* < .001). No significant difference in gender distribution was observed (*P* = .090). Laboratory parameters: non-survivors had significantly higher RDW, anion gap, potassium, PT, partial thromboplastin time, INR, BUN, and creatinine levels (all *P* < .001), and significantly lower RBC count, hemoglobin, chloride, lymphocyte count, and albumin levels (all *P* < .001). Platelet count did not differ significantly between groups (*P* = .098). Disease severity: non-survivors had significantly higher SOFA, APS III, and OASIS scores (all *P* < .001). The distribution of SIRS scores also differed significantly (*P* < .001), with non-survivors having a higher proportion of scores 3 and 4. Comorbidities: non-survivors had a significantly higher prevalence of atrial fibrillation, myocardial infarction, cerebrovascular disease, liver disease, renal disease, malignant cancer, and coronary artery disease (all *P* < .05). Conversely, survivors had a significantly higher prevalence of hypertension (*P* < .001). Prevalence of diabetes mellitus, dyslipidemia, COPD, and cardiomyopathy did not differ significantly between survivors and non-survivors (*P* = .11, *P* = .558, *P* = .15, *P* = .087 respectively). Interventions: non-survivors had significantly lower use of diuretics, antiplatelet agents, statins, ACEIs/ARBs, implantable cardioverter defibrillators/pacemakers, PCI, and CABG (all *P* < .05). Use of anticoagulants did not differ significantly (*P* = .449).

**Table 2:** Baseline characteristics of the survival and non-survival groups.

	Total (n = 7620)	Survival (n = 6399)	Non-survival (n = 1221)	
Age, yr (mean (SD))	71.96 (13.67)	71.09 (13.80)	76.48 (11.97)	<.001
Gender, n (%)				
Male	4172 (54.8)	3476 (54.3)	696 (57.0)	
Female	3448 (45.2)	2923 (45.7)	525 (43.0)	.09
Length of stay (LOS, d)	15.32 (13.89)	15.91 (14.53)	12.26 (9.28)	<.001
ICU length of stay (ICULOS, d)	5.35 (6.10)	5.17 (6.26)	6.26 (5.10)	<.001
WBC (K/μL)	12.29 (8.08)	12.04 (7.83)	13.61 (9.18)	<.001
RBC (m/μL)	3.54 (0.80)	3.57 (0.81)	3.43 (0.79)	<.001
Platelet (K/μL)	217.59 (99.69)	218.41 (97.86)	213.26 (108.72)	.098
RDW (%)	15.98 (2.48)	15.82 (2.39)	16.84 (2.74)	<.001
Hemoglobin (g/dL)	10.37 (2.49)	10.44 (2.53)	9.98 (2.20)	<.001
Sodium (mmol/L)	137.32 (4.37)	137.34 (4.30)	137.23 (4.70)	.394
Potassium (mmol/L)	4.25 (0.60)	4.24 (0.59)	4.30 (0.62)	.002
Chloride (mmol/L)	100.82 (6.50)	100.95 (6.39)	100.11 (6.98)	<.001
Aniongap (mmol/L)	15.26 (4.11)	15.04 (3.95)	16.39 (4.68)	<.001
PT (s)	18.49 (10.58)	17.96 (9.85)	21.29 (13.42)	<.001
PTT (s)	36.41 (12.90)	36.13 (12.78)	37.87 (13.44)	<.001
INR	1.62 (0.78)	1.59 (0.75)	1.79 (0.89)	<.001
Lymphocyte count (K/μL)	12.24 (9.22)	12.97 (9.26)	8.46 (7.99)	<.001
BUN (mg/dL)	38.53 (26.40)	36.65 (25.21)	48.36 (30.05)	<.001
CRE (mg/dL)	1.91 (1.62)	1.86 (1.62)	2.18 (1.63)	<.001
Albumin (g/dL)	3.24 (0.60)	3.29 (0.59)	2.96 (0.58)	<.001
SOFA	5.50 (3.28)	5.19 (3.13)	7.08 (3.57)	<.001
APSIII	50.00 (18.17)	47.99 (17.03)	60.57 (20.22)	<.001
OASIS	32.66 (8.40)	31.95 (8.17)	36.41 (8.62)	<.001
SIRS, n (%)				
0	44 (0.6)	41 (0.6)	3 (0.2)	<.001
1	895 (11.7)	796 (12.4)	99 (8.1)	
2	2614 (34.3)	2263 (35.4)	351 (28.7)	
3	3002 (39.4)	2481 (38.8)	521 (42.7)	
4	1065 (14.0)	818 (12.8)	247 (20.2)	
Hypertension, n (%)	1588 (20.8)	1383 (21.6)	205 (16.8)	<.001
Diabetes, n (%)	3501 (45.9)	2966 (46.4)	535 (43.8)	.11
Atrial fibrillation, n (%)	4173 (54.8)	3384 (52.9)	789 (64.6)	<.001
Dyslipidemia, n (%)	3700 (48.6)	3117 (48.7)	583 (47.7)	.558
Myocardial infarct, n (%)	2746 (36.0)	2229 (34.8)	517 (42.3)	<.001
Cerebrovascular disease, n (%)	908 (11.9)	722 (11.3)	186 (15.2)	<.001
COPD, n (%)	2958 (38.8)	2507 (39.2)	451 (36.9)	.15
Liver disease, n (%)	829 (10.9)	645 (10.1)	184 (15.1)	<.001
Renal disease, n (%)	3547 (46.5)	2910 (45.5)	637 (52.2)	<.001
Malignant cancer, n (%)	808 (10.6)	597 (9.3)	211 (17.3)	<.001
Coronary artery disease, n (%)	3540 (46.5)	2935 (45.9)	605 (49.5)	.02
Cardiomyopathy, n (%)	1817 (23.8)	1502 (23.5)	315 (25.8)	.087
Implantable cardioverter defibrillator pacemaker, n (%)	414 (5.4)	382 (6.0)	32 (2.6)	<.001
Percutaneous coronary intervention, n (%)	915 (12.0)	804 (12.6)	111 (9.1)	.001
Coronary bypass operation, n (%)	532 (7.0)	504 (7.9)	28 (2.3)	<.001
Used diuretic, n (%)	6965 (91.4)	5891 (92.1)	1074 (88.0)	<.001
Used antiplatelet, n (%)	5502 (72.2)	4671 (73.0)	831 (68.1)	<.001
Used anticoagulant, n (%)	7256 (95.2)	6099 (95.3)	1157 (94.8)	.449
Used statin, n (%)	4921 (64.6)	4176 (65.3)	745 (61.0)	.005
Used ACEI/ARB, n (%)	3459 (45.4)	3213 (50.2)	246 (20.1)	<.001
CAR (mean (SD))	0.61 (0.55)	0.59 (0.53)	0.76 (0.61)	<.001

Continuous variables are presented as mean ± standard deviation (SD), and categorical variables are expressed as number (%).

APS III = Acute Physiology Score III; BUN = blood urea nitrogen; CAR = creatinine-to-albumin ratio; COPD = chronic obstructive pulmonary disease; INR = international normalized ratio; OASIS = Oxford Acute Severity of Illness Score; PT = prothrombin time; PTT = partial thromboplastin time; RBC = red blood cell count; RDW = red blood cell distribution width; SIRS = Systemic Inflammatory Response Syndrome; SD = standard deviation; SOFA = Sequential Organ Failure Assessment; WBC = white blood cell count.

### 3.2. Relationship between CAR and mortality risk in AHF patients

**Figure 2.**
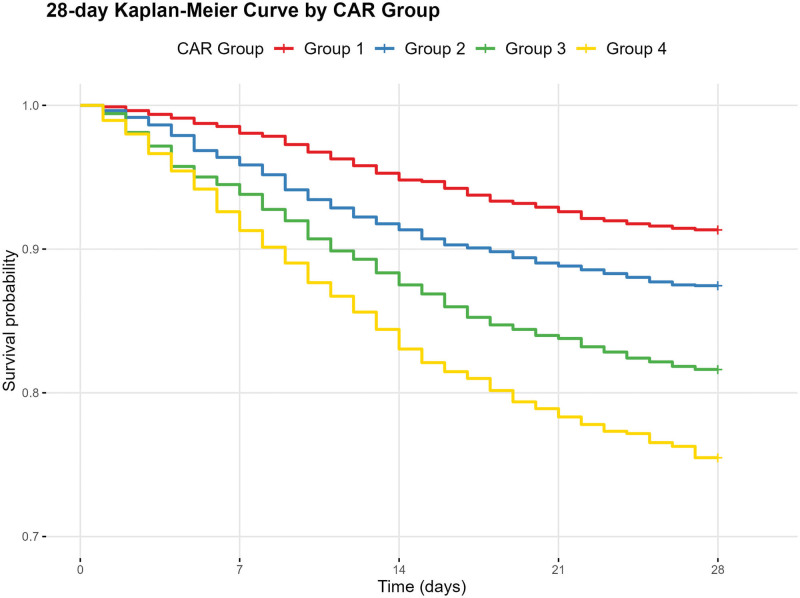
Kaplan–Meier survival curves by creatinine-to-albumin ratio (CAR) quartiles. Kaplan–Meier survival curves showing the association between CAR quartiles and 28-day all-cause mortality in patients with acute heart failure (AHF). Patients in higher CAR quartiles exhibited significantly lower survival probabilities compared with those in the lowest quartile (log-rank *P* < .001).

Kaplan–Meier analysis results showed significant differences in 28-day all-cause mortality rates between CAR groups (log-rank *P* < .001). Specifically, Group 4 (the group with the highest CAR levels) had a significantly higher mortality rate within 28 days than other groups, while Group 1 had the lowest mortality rate. Figure 2 shows the survival curves for different CAR groups, with Group 4’s survival curve significantly lower than Group 1’s, demonstrating a clear gradient difference, further confirming the positive correlation between CAR levels and short-term mortality risk in AHF patients. Notably, similar statistical significance was observed at the 180-day, and 365-day mortality endpoints (Figures S1 and S2, Supplemental Digital Content, https://links.lww.com/MD/R10), further reinforcing the potential of CAR as a prognostic marker for mortality in patients with AHF.

**Figure 3.**
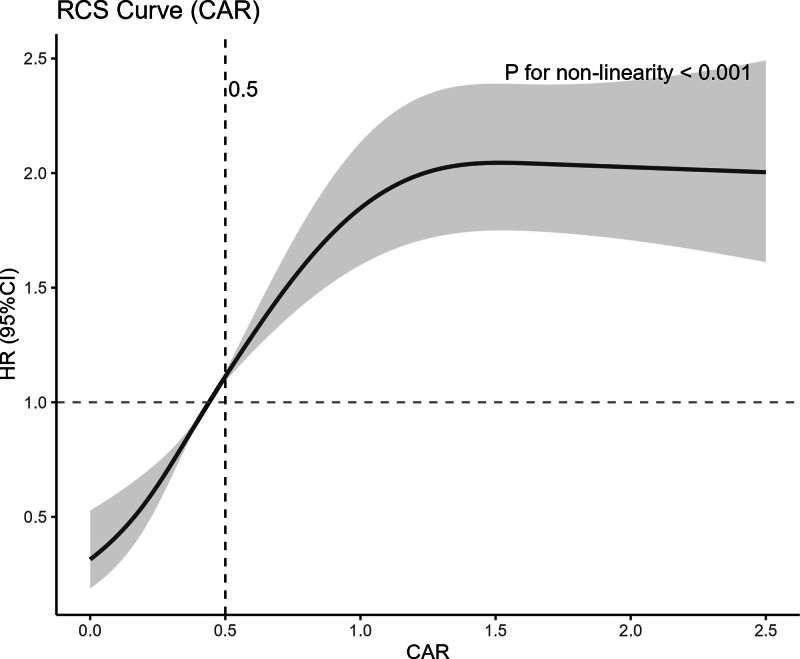
Dose–response relationship between creatinine-to-albumin ratio (CAR) and 28-day all-cause mortality. Restricted cubic spline (RCS) curves illustrating the nonlinear association between CAR and 28-day all-cause mortality in patients with acute heart failure (AHF). The RCS model was adjusted for age, sex, coronary artery disease, diabetes, COPD, myocardial infarction, atrial fibrillation, cardiomyopathy, chronic kidney disease, stroke, dyslipidemia, hypertension, liver disease, malignant tumors, creatinine, potassium, sodium, hemoglobin, platelets, WBC, red blood cell distribution width (RDW), diuretics, ACEIs/ARBs, statins, antiplatelet agents, anticoagulants, CABG/PCI, and implantable cardioverter-defibrillator (ICD) pacemaker implantation (*P* for nonlinearity < .001). ACEIs = angiotensin-converting enzyme inhibitors, ARBs = angiotensin receptor blockers, CABG = coronary artery bypass grafting, COPD = chronic obstructive pulmonary disease, PCI = percutaneous coronary intervention.

The results of the multivariate Cox regression analysis (Table 3) show significant differences in mortality risk between different CAR groups. The 28-day mortality risk in Group 2 was significantly higher than that in Group 1 (hazard ratio [HR] = 1.492, 95% confidence interval [CI]: 1.223–1.819; *P* < .01), indicating that even a mild increase in CAR levels significantly increases the mortality risk in AHF patients. The risk further increased in Group 3 (HR = 2.260, 95% CI: 1.878–2.719; *P* < .01), while the mortality risk in Group 4 significantly increased to HR = 3.123 (95% CI: 2.615–3.729; *P* < .01), indicating a dose–response relationship between elevated CAR levels and mortality risk. These findings suggest that elevated CAR levels are not only predictive of short-term mortality risk but also exhibit a similar trend in long-term mortality risk prediction (e.g., 180 days, 365 days) (Tables S1 and S2, Supplemental Digital Content, https://links.lww.com/MD/R10). RCS modeling further validated the continuous relationship between CAR and mortality risk (see Fig. 3). After adjusting for covariates such as age, gender, and comorbidities, the RCS curve showed a smooth, monotonically increasing association between CAR levels and 28-day mortality risk. As CAR levels increased, the mortality risk in AHF patients gradually rose, consistent with the results of Cox regression analysis, further supporting CAR as an important biomarker for predicting mortality risk in AHF patients. Importantly, the observed associations between CAR subgroups and mortality risk were consistent across all adjusted models, further supporting the robustness of the findings. In addition, restricted cubic spline analyses for 180- and 365-day all-cause mortality also demonstrated similar nonlinear associations (Figures S3 and S4, Supplemental Digital Content, https://links.lww.com/MD/R10).

**Table 3:** Association between creatinine-to-albumin ratio (CAR) quartiles and 28-day all-cause mortality.

Group	Model 1_HR_CI	Model 1_*P*	Model 2_HR_CI	Model 2_*P*	Model 3_HR_CI	Model 3_*P*	Model 4_HR_CI	Model 4_*P*
Group 1	REF	REF	REF	REF	REF	REF	REF	REF
Group 2	1.492 (1.223–1.819)	<.01	1.382 (1.132–1.688)	<.01	1.497 (1.223–1.832)	<.01	1.441 (1.175–1.767)	<.01
Group 3	2.260 (1.878–2.719)	<.01	2.045 (1.696–2.466)	<.01	2.433 (1.994–2.969)	<.01	2.119 (1.722–2.609)	<.01
Group 4	3.123 (2.615–3.729)	<.01	3.122 (2.606–3.739)	<.01	4.015 (3.268–4.933)	<.01	3.360 (2.605–4.335)	<.01

Model 1: unadjusted.

Model 2: adjusted for age and sex.

Model 3: adjusted for age, sex, coronary heart disease, diabetes, chronic obstructive pulmonary disease (COPD), myocardial infarction, atrial fibrillation, cardiomyopathy, chronic kidney disease, stroke, dyslipidemia, hypertension, liver disease, and malignant tumors.

Model 4: adjusted for all variables in Model 3 plus creatinine, potassium, sodium, hemoglobin, platelets, white blood cells (WBC), red blood cell distribution width (RDW), diuretics, angiotensin-converting enzyme inhibitors (ACEIs), angiotensin receptor blockers (ARBs), statins, antiplatelet agents, anticoagulants, coronary artery bypass grafting (CABG), percutaneous coronary intervention (PCI), and implantable cardioverter-defibrillator (ICD) pacemaker implantation.

CAR = creatinine-to-albumin ratio, CI = confidence interval, HR = hazard ratio, Ref = reference value.

### 3.3. Subgroup analyses

**Figure 4.**
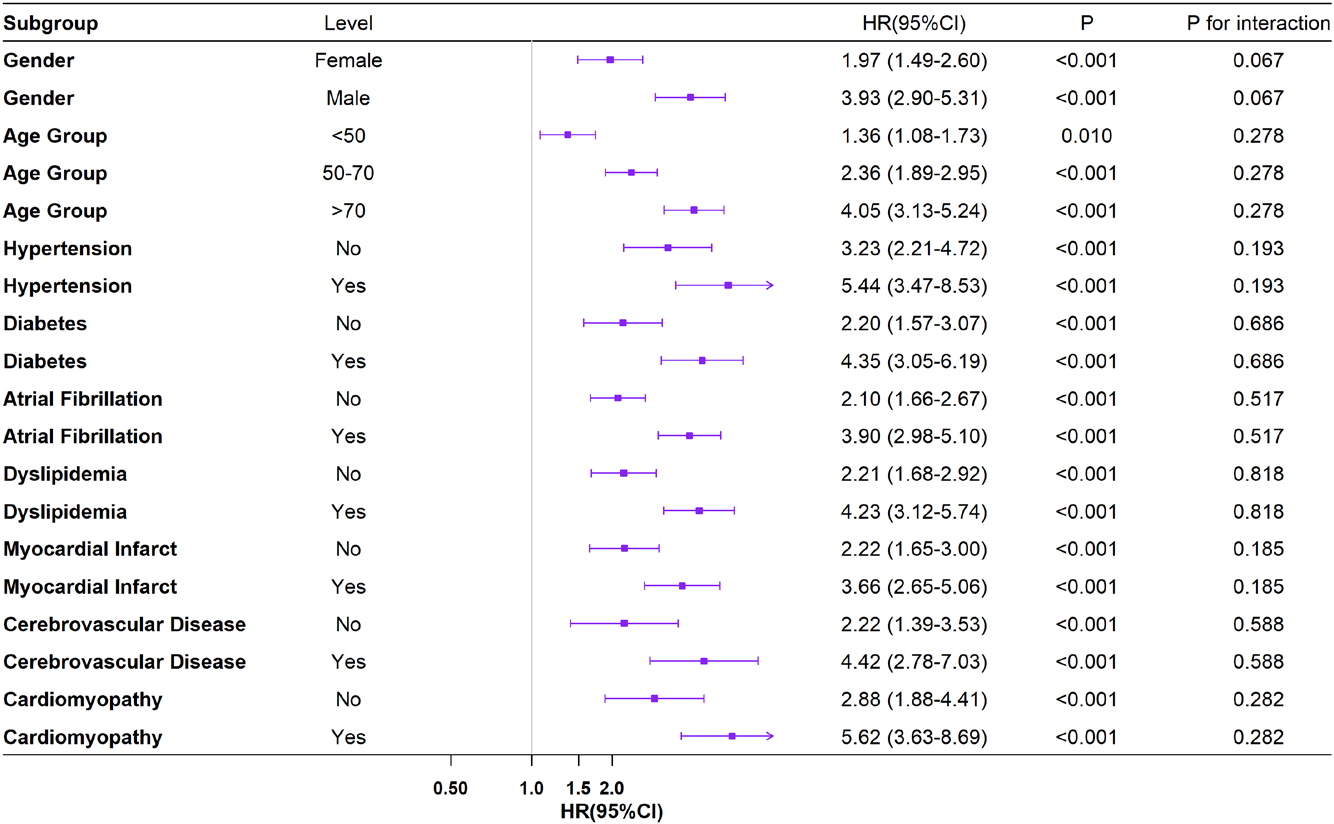
Subgroup analysis of the association between creatinine-to-albumin ratio (CAR) and 28-day all-cause mortality. Forest plots showing hazard ratios (HRs) and 95% confidence intervals (CIs) for the association between CAR and 28-day all-cause mortality across clinically relevant subgroups, including sex, age groups, comorbidities, and cardiovascular risk factors. No significant interaction effects were observed across most subgroups.

To further probe the prognostic significance of CAR across diverse populations, we performed stratified analyses according to various clinical features. The findings revealed that the link between CAR and mortality risk persisted in all subgroups. However, there was some variability in the HR values among the subgroups. For the age-stratified analysis, as shown in Figure 4, in the < 50 years age group, the HR was 1.36 (95% CI: 1.08–1.73) with a *P*-value of .010. In the 50 to 70 years age group, the HR was 2.36 (95% CI: 1.89–2.95) and *P* < .001. For those > 70 years, the HR was 4.05 (95% CI: 3.13–5.24) and *P* < .001. The *P* for interaction for age group was .278, which was non-significant, implying that although HRs differed across age groups, age did not significantly alter the association between CAR and 28-day mortality risk.

In the analysis of comorbidities, for hypertension, non-hypertensive patients had an HR of 3.23 (95% CI: 2.21–4.72, *P* < .001), while hypertensive patients had an HR of 5.44 (95% CI: 3.47–8.53, *P* < .001), with a *P* for interaction of .193, indicating hypertension did not significantly modify the CAR–mortality risk relationship. For diabetes, non-diabetic patients had an HR of 2.20 (95% CI: 1.57–3.07, *P* < .001), and diabetic patients had an HR of 4.35 (95% CI: 3.05–6.19, *P* < .001), with a *P* for interaction of .686, confirming CAR as an independent mortality risk predictor in AHF. In patients with atrial fibrillation, the HR was 3.90 (95% CI: 2.98–5.10, *P* < .001), compared to 2.10 (95% CI: 1.66–2.67, *P* < .001) in those without, and the *P* for interaction was .517, suggesting atrial fibrillation did not significantly affect the relationship. Similarly, for myocardial infarction, cerebrovascular disease, and cardiomyopathy, the *P* for interaction values (.185, .588, and .282, respectively) were non-significant, indicating these comorbidities did not notably impact the prognostic role of CAR, thus supporting CAR’s universal applicability as a prognostic assessment tool for AHF patients.

## 4. Discussion

This study is the 1st to assess the prognostic value of the CAR in patients with AHF through a retrospective cohort analysis. The results indicate a significant dose–response relationship between CAR and the risk of death in patients with AHF. By analyzing data from 7620 AHF patients in the MIMIC-IV database, we found that patients with higher CAR values (the 3rd quartile above 0.72) had a significantly increased risk of 28-day all-cause mortality compared to those with lower CAR values (the 3rd quartile below 0.30), with a HR of 3.123 (95% CI: 2.615–3.729).^[13–15]^ This finding suggests that CAR not only serves as a short-term prognostic predictor for AHF but also provides an effective warning for long-term mortality risk, offering clinicians a new, convenient tool for identifying high-risk patients.

Compared with traditional biomarkers and risk stratification tools, the C-reactive protein-to-albumin ratio provides complementary prognostic value in critically ill patients with AHF. A study using the UK Biobank cohort demonstrated that CAR is significantly associated with cardiovascular events and mortality risk in the general population.^[16]^ Importantly, a retrospective study specifically targeting AHF patients showed that a higher CAR at admission independently predicted increased in-hospital mortality.^[17]^ In contrast, cardiac troponin, as a marker of myocardial injury, remains a well-established predictor of both short- and long-term mortality in hospitalized AHF patients.^[18]^ Taken together, CAR integrates inflammatory, nutritional, and renal functional domains, thereby complementing conventional biomarkers such as BNP/NT-proBNP and cardiac troponin, as well as risk stratification scores like Acute Decompensated Heart Failure National Registry and Organized Program to Initiate Lifesaving Treatment in Hospitalized Patients with Heart Failure, which may have limited prognostic applicability in intensive care settings.

Beyond these comparative and therapeutic insights, the mechanistic implications of CAR in AHF pathophysiology are also noteworthy, as discussed below. AHF is a clinical syndrome caused by multiple pathological processes, characterized by impaired cardiac function, inadequate tissue perfusion, and metabolic abnormalities. The role of the kidneys in HF has long been recognized, with renal dysfunction commonly observed in AHF patients and closely associated with the course of HF.^[19,20]^ Renal damage leading to tubular and interstitial injury not only exacerbates the symptoms of HF but also increases the risk of mortality in patients. Hypoalbuminemia is closely associated with malnutrition, fluid retention, and systemic inflammatory response in HF patients. A decrease in albumin levels typically reflects the deterioration of the patient’s overall nutritional status and inflammatory response.^[15,21]^ CAR, as a biomarker that combines renal function and nutritional status, can effectively reflect the combined changes in these 2 key factors, making its prognostic value in patients with AHF worthy of further exploration.

Through analysis, we found that elevated CAR levels may be closely associated with worsening myocardial injury and exacerbated systemic inflammatory responses in patients with AHF. Acute episodes of AHF are often accompanied by the release of a large number of cytokines and activation of the immune system, which can lead to further myocardial damage and ventricular remodeling. Elevated CAR levels reflect impaired renal function and the presence of hypoalbuminemia, and these pathological states themselves can trigger and exacerbate inflammatory responses, oxidative stress, and apoptosis, thereby accelerating the progression of HF.^[13,22–24]^ Additionally, elevated CAR levels may further exacerbate cardiac burden by affecting fluid balance, worsening edema, and increasing pulmonary congestion. Therefore, CAR not only serves as an indicator of renal function and nutritional status but may also provide deeper insights into the physiological and pathological processes of HF, offering additional information about the patient’s condition.

The immune system of patients with AHF is often in a state of excessive activation during the inflammatory response, and these changes in immune response and excessive cytokine release play a significant role in the clinical manifestations of HF.^[22,23]^ For example, elevated levels of pro-inflammatory cytokines such as interleukin-6 and tumor necrosis factor-alpha have been shown to be closely associated with clinical outcomes in patients with AHF.^[25,26]^ These cytokines not only promote myocardial damage but also participate in the process of cardiac remodeling, further exacerbating HF symptoms.^[27–29]^ As these immune responses worsen, the treatment of HF becomes more complex, and therefore effective immune modulation strategies are expected to become a new direction for the future treatment of AHF.

Compared to traditional single biomarkers, the advantage of CAR lies in its ability to simultaneously reflect 2 important physiological processes: renal function and nutritional status. These 2 factors play a crucial role in the pathogenesis of AHF. Single biomarkers such as C-reactive protein or WBC count can reflect inflammatory responses, but they typically fail to provide information on renal function or systemic nutritional status, limiting their utility in prognostic assessment for complex diseases.^[30]^ CAR, by integrating the effects of renal function and nutritional status, offers clinicians a comprehensive and sensitive prognostic assessment tool.^[31]^ Through this multidimensional assessment, CAR demonstrates superior prognostic accuracy compared to traditional markers in predicting mortality risk in patients with AHF.^[13]^

Our findings may also have potential therapeutic implications. Hypoalbuminemia is common in ICU-admitted AHF patients, and while albumin supplementation remains debated, some evidence suggests it may reduce short-term mortality in specific subpopulations. A recent propensity-matched cohort study showed that AHF patients receiving albumin had significantly lower 30-day mortality (HR ≈ 0.53).^[32]^ These findings suggest that patients with elevated CAR (indicating low albumin and impaired renal function) may benefit from tailored interventions, such as targeted albumin administration, intensified decongestive strategies, or early initiation of guideline-directed therapy.

Several limitations should be acknowledged. First, only ICU-admitted AHF patients were included, which may limit the generalizability of our findings to less critically ill populations. Second, our endpoints were restricted to all-cause mortality; important patient-centered outcomes (e.g., rehospitalization, functional status, quality of life) were not assessed.^[33]^ Third, CAR was measured only at ICU admission, without capturing dynamic trends during hospitalization. Fourth, data were derived from a single-center, U.S. database (MIMIC-IV), so residual confounding may persist despite multivariate adjustment. These limitations should be considered when interpreting our findings, and they highlight the need for further validation. Finally, the observational nature of the study precludes causal inference regarding CAR and outcomes. Future multicenter prospective studies are needed to validate CAR’s utility, explore its combination with biomarkers like NT-proBNP, and assess its potential as a personalized treatment marker.

Dynamic changes in the CAR may also provide valuable clinical information. Serial monitoring of CAR could help clinicians assess disease trajectory and guide real-time treatment adjustments in AHF. For example, decreases in CAR may reflect reduced inflammation or effective decongestion, whereas persistently elevated levels could suggest ongoing disease progression or complications. This highlights the potential utility of CAR not only as a baseline prognostic marker but also as a dynamic biomarker for monitoring treatment response. Future multicenter prospective studies incorporating serial CAR measurements will be crucial to determine whether dynamic changes can guide individualized treatment strategies in AHF.

## 5. Conclusions

CAR, as a simple, easily accessible, and cost-effective biomarker, holds significant potential for application in the clinical management of patients with AHF. It can comprehensively assess kidney function, nutritional status, and systemic inflammatory responses, providing additional dimensions of information for patient risk assessment. Through dynamic monitoring of CAR, clinicians can adjust treatment strategies in a timely manner based on changes in patient condition, further enhancing treatment outcomes. Future research will further explore the potential of combining CAR with other biomarkers to provide more precise and personalized treatment plans for patients with AHF.

## Author contributions

**Conceptualization:** Yuanxi Feng.

**Data curation:** Shenglan Liu, Chengyu Zhang, Yuanxi Feng.

**Formal analysis:** Chengyu Zhang.

**Funding acquisition:** Yuanxi Feng.

**Investigation:** Shenglan Liu, Yuanxi Feng.

**Methodology:** Shenglan Liu, Chengyu Zhang, Yuanxi Feng.

**Project administration:** Shenglan Liu, Chengyu Zhang.

**Software:** Shenglan Liu.

**Validation:** Shenglan Liu, Yuanxi Feng.

**Visualization:** Shenglan Liu.

**Writing – original draft:** Shenglan Liu, Yuanxi Feng.

**Writing – review & editing:** Shenglan Liu, Yuanxi Feng.

## Supplementary Material


