# Supplementary material for: The relationship between serum creatinine-to-albumin ratio (CAR) and prognosis in critically ill patients with acute heart failure admitted to the ICU: A retrospective study based on the MIMIC-IV database
Source: Medicine (Baltimore). 2026 Jan 9;105(2):e46825. doi: 10.1097/MD.0000000000046825 (PMC12794988; doi:10.1097/MD.0000000000046825)
Supplement: Supplementary file 1 [file medi-105-e46825-s001.pdf]

Supplementary Figure 1. Kaplan–Meier Survival Curves for 180-Day All-Cause Mortality by CAR Quartiles. Kaplan–Meier survival curves illustrating the association between creatinine-to-albumin ratio (CAR) quartiles and 180-day all-cause mortality among patients with acute heart failure (AHF). Patients in higher CAR quartiles had significantly lower survival probabilities compared with those in the lowest quartile (log-rank P < 0.001).

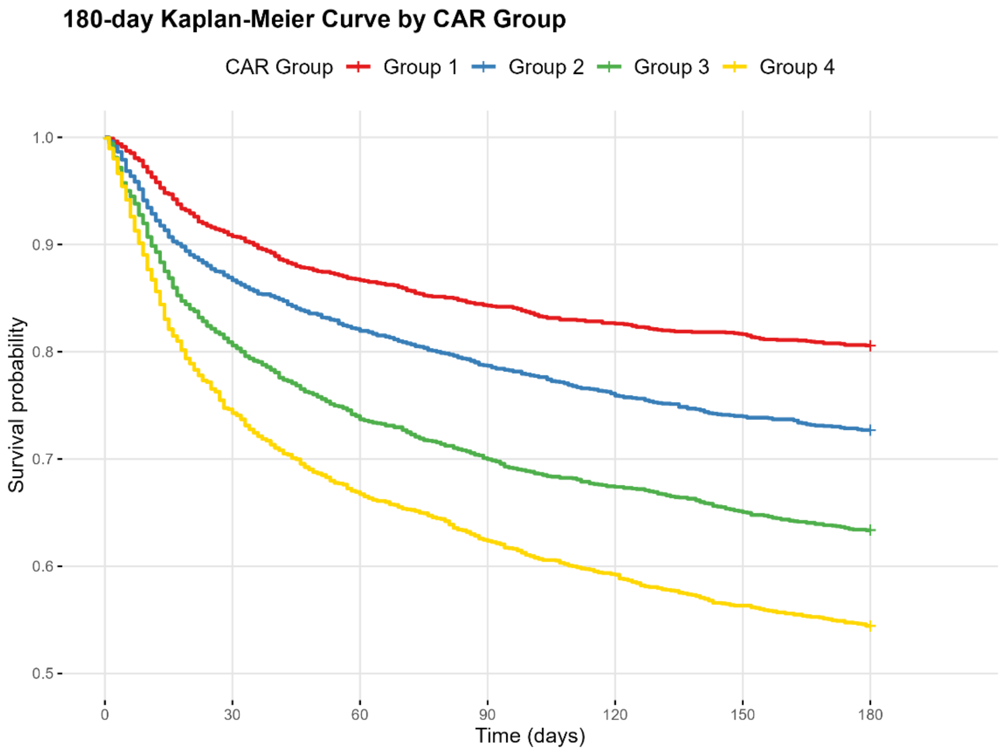

Supplementary Figure 2. Kaplan–Meier Survival Curves for 365-Day All-Cause Mortality by CAR Quartiles. Kaplan–Meier survival curves demonstrating the association between CAR quartiles and 365-day all-cause mortality in patients with AHF. A clear dose-response gradient was observed, with the highest CAR quartile showing the lowest survival probability (log-rank P < 0.001).

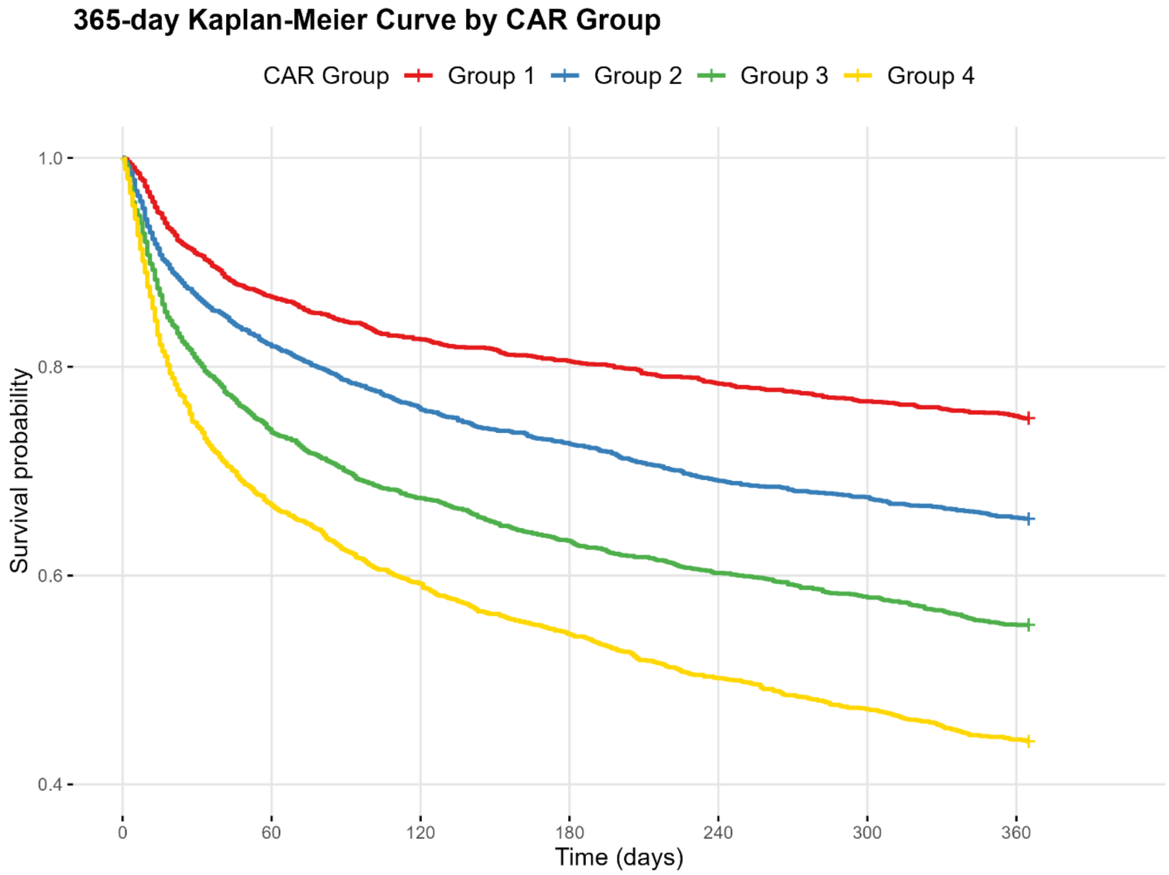

Supplementary Figure 3. Dose-Response Relationship Between CAR and 180-Day All-Cause Mortality. Restricted cubic spline (RCS) curves showing the nonlinear association between CAR and 180-day all-cause mortality in patients with AHF. The RCS model was adjusted for age, sex, coronary artery disease, diabetes, chronic obstructive pulmonary disease (COPD), myocardial infarction, atrial fibrillation, cardiomyopathy, chronic kidney disease, stroke, dyslipidemia, hypertension, liver disease, malignant tumors, creatinine, potassium, sodium, hemoglobin, platelets, white blood cell count (WBC), red blood cell distribution width (RDW), diuretics, angiotensin-converting enzyme inhibitors (ACEIs), angiotensin receptor blockers (ARBs), statins, antiplatelet agents, anticoagulants, coronary artery bypass grafting (CABG), percutaneous coronary intervention (PCI), and implantable cardioverter-defibrillator (ICD) pacemaker implantation. P for nonlinearity < 0.001.

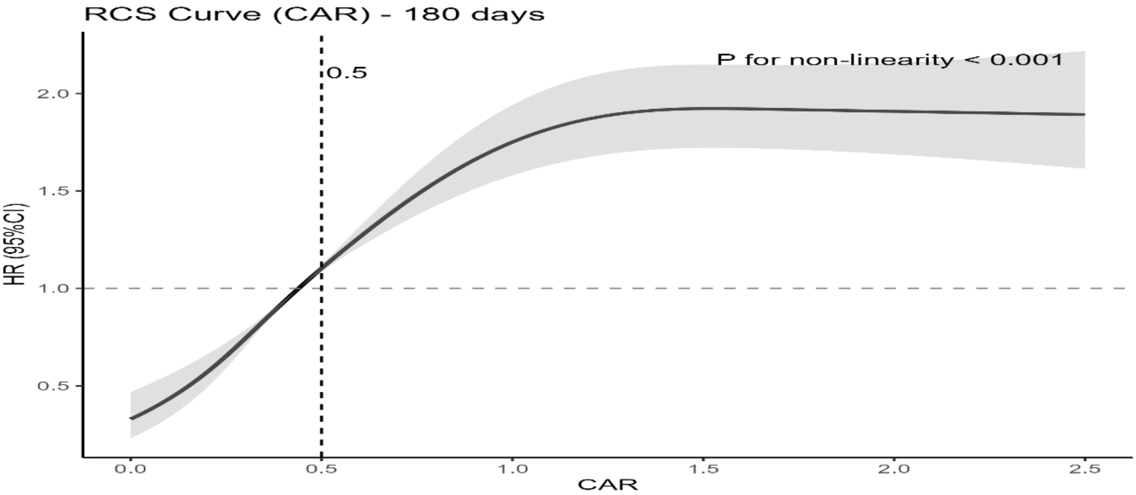

Supplementary Figure 4. Dose-Response Relationship Between CAR and 365-Day All-Cause Mortality. Restricted cubic spline (RCS) curves showing the nonlinear relationship between CAR and 365-day all-cause mortality in patients with AHF. The RCS model was adjusted for the same covariates as Supplementary Figure 3. P for nonlinearity < 0.001.

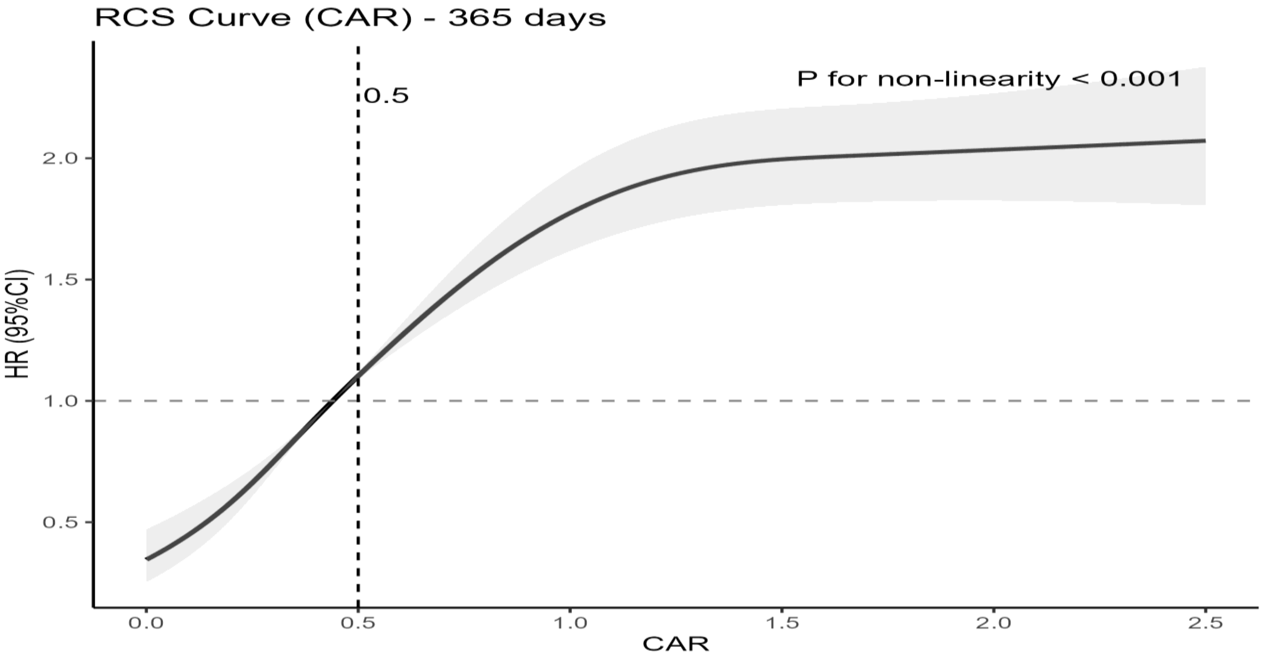

Group	Model1_HR_CI	Model1_P	Model2_HR_CI	Model2_P	Model3_HR_CI	Model3_P	Model4_HR_CI	Model4_P
group1	REF	REF	REF	REF	REF	REF	REF	REF
group2	1.475(1.291,1.685)	<0.01	1.366(1.194,1.563)	<0.01	1.417(1.236,1.625)	<0.01	1.352(1.177,1.553)	<0.01
group3	2.125(1.873,2.410)	<0.01	1.932(1.701,2.195)	<0.01	2.077(1.809,2.385)	<0.01	1.838(1.591,2.124)	<0.01
group4	2.840(2.514,3.208)	<0.01	2.874(2.539,3.253)	<0.01	3.190(2.763,3.683)	<0.01	2.811(2.348,3.364)	<0.01

Supplementary Table 1. Association Between Creatinine-to-Albumin Ratio (CAR) Quartiles and 180-Day All-Cause Mortality. Model 1: Unadjusted. Model 2: Adjusted for age and sex. Model 3: Adjusted for age, sex, coronary heart disease, diabetes, COPD, myocardial infarction, atrial fibrillation, cardiomyopathy, chronic kidney disease, stroke, dyslipidemia, hypertension, liver disease, and malignant tumors. Model 4: Adjusted for all variables in Model 3 plus creatinine, potassium, sodium, hemoglobin, platelets, WBC, RDW, diuretics, ACEIs, ARBs, statins, antiplatelet agents, anticoagulants, CABG, PCI, and ICD pacemaker implantation. Abbreviations: HR, hazard ratio; CI, confidence interval; Ref, reference value; CAR, creatinine-to-albumin ratio.

Group	Model1_HR_CI	Model1_P	Model2_HR_CI	Model2_P	Model3_HR_CI	Model3_P	Model4_HR_CI	Model4_P
group1	REF	REF	REF	REF	REF	REF	REF	REF
group2	1.477(1.313,1.663)	<0.01	1.390(1.234,1.566)	<0.01	1.398(1.238,1.578)	<0.01	1.320(1.167,1.493)	<0.01
group3	2.085(1.863,2.332)	<0.01	1.931(1.724,2.164)	<0.01	1.950(1.722,2.207)	<0.01	1.694(1.488,1.928)	<0.01
group4	2.860(2.566,3.187)	<0.01	2.949(2.641,3.292)	<0.01	3.005(2.642,3.417)	<0.01	2.448(2.087,2.871)	<0.01

Supplementary Table 2. Association Between Creatinine-to-Albumin Ratio (CAR) Quartiles and 365-Day All-Cause Mortality. Model 1: Unadjusted. Model 2: Adjusted for age and sex. Model 3: Adjusted for age, sex, coronary heart disease, diabetes, COPD, myocardial infarction, atrial fibrillation, cardiomyopathy, chronic kidney disease, stroke, dyslipidemia, hypertension, liver disease, and malignant tumors. Model 4: Adjusted for all variables in Model 3 plus creatinine, potassium, sodium, hemoglobin, platelets, WBC, RDW, diuretics, ACEIs, ARBs, statins, antiplatelet agents, anticoagulants, CABG, PCI, and ICD pacemaker implantation. Abbreviations: HR, hazard ratio; CI, confidence interval; Ref, reference value; CAR, creatinine-to-albumin ratio.
